# Making hosts smell tastier: How mosquito‐borne viruses take the initiative in viral transmission

**DOI:** 10.1002/ctm2.1168

**Published:** 2023-01-07

**Authors:** Yibin Zhu, Gong Cheng

**Affiliations:** ^1^ Tsinghua University‐Peking University Joint Center for Life Sciences, School of Medicine Tsinghua University Beijing China; ^2^ Shenzhen Bay Laboratory Institute of Infectious Diseases Shenzhen Guangdong China

1

There are hundreds of mosquito‐borne viruses in nature that can be carried and transmitted by mosquitoes to human and animal hosts, causing serious diseases such as viral encephalitis, meningitis and haemorrhagic fever. Over the past two decades, emerging and re‐emerging mosquito‐borne viruses, including dengue virus, Zika virus, chikungunya virus and West Nile virus, have become a public health challenge worldwide, causing billions of infections and hundreds of thousands of deaths each year.^1^ For instance, approximately 2.5 billion people in the world live in areas where dengue virus is endemic. Each year, approximately 390 million people become infected or reinfected with dengue virus, leading to 500 000–1 million hospital admissions, and dengue fever epidemics have occurred in more than 100 countries and regions worldwide.^2^ Mosquito‐borne viruses are transmitted from mosquitoes to hosts. In the virus transmission cycle, mosquitoes need to find, locate and bite an infected person or animal to feed on the blood that carries the virus. Subsequently, mosquitoes are able to carry and rapidly transmit the virus.^3^

If a mosquito bites an uninfected person, it cannot become infected, and the virus transmission cycle is interrupted. At the beginning of a mosquito‐borne viral epidemic, the proportion of infected individuals in the population is not high. Instead, mosquitoes are able to select infected individuals in the population to bite, thus accelerating the spread of the virus and causing an epidemic outbreak. How effectively the mosquito locates the infected host and acquires the virus is the main rate‐limiting step in the ‘host‐virus‐mosquito’ transmission cycle. How do mosquitoes locate their infectors, and what are the reasons for the rapid spread of mosquito‐borne viruses in nature? In a recent study published in *Cell*, Zhang et al. identified a previously unknown role of dengue and Zika viruses in regulating the host to promote viral transmission.^4^ The authors built two classical behavioural devices (a three‐cage olfactometry device and a two‐arm olfactometry device) and found that mice infected with dengue and Zika viruses were significantly more attractive to *Aedes aegypti* and *Aedes albopictus*. The authors then analysed the body temperature, carbon dioxide release and volatile odour of virus‐infected mice and found that altered host odour was a determining factor in attracting mosquitoes to infected hosts. Further results showed that mice released a volatile compound, acetophenone, in large quantities after mosquito‐borne virus infection and that acetophenone effectively activated the olfactory nervous system of mosquitoes, enhancing their behavioural tendencies towards infected mice.^4^

The authors then collected odour samples from dengue patients and healthy volunteers. Their data revealed that the odours from dengue patients were a stronger attraction for *A. aegypti* mosquitoes. Moreover, the odours of dengue patients also contained significantly higher levels of acetophenone than those of healthy volunteers. Consistently, different concentrations of acetophenone applied to human arms for a mosquito behavioural assay demonstrated a more potent mosquito‐attracting effect in a dose‐dependent manner. The authors’ study suggests that dengue patients significantly increase their attractiveness to mosquitoes by releasing large amounts of acetophenone to alter their own odour, thereby accelerating the spread of the virus^4^ (Figure 1).

**FIGURE 1:**
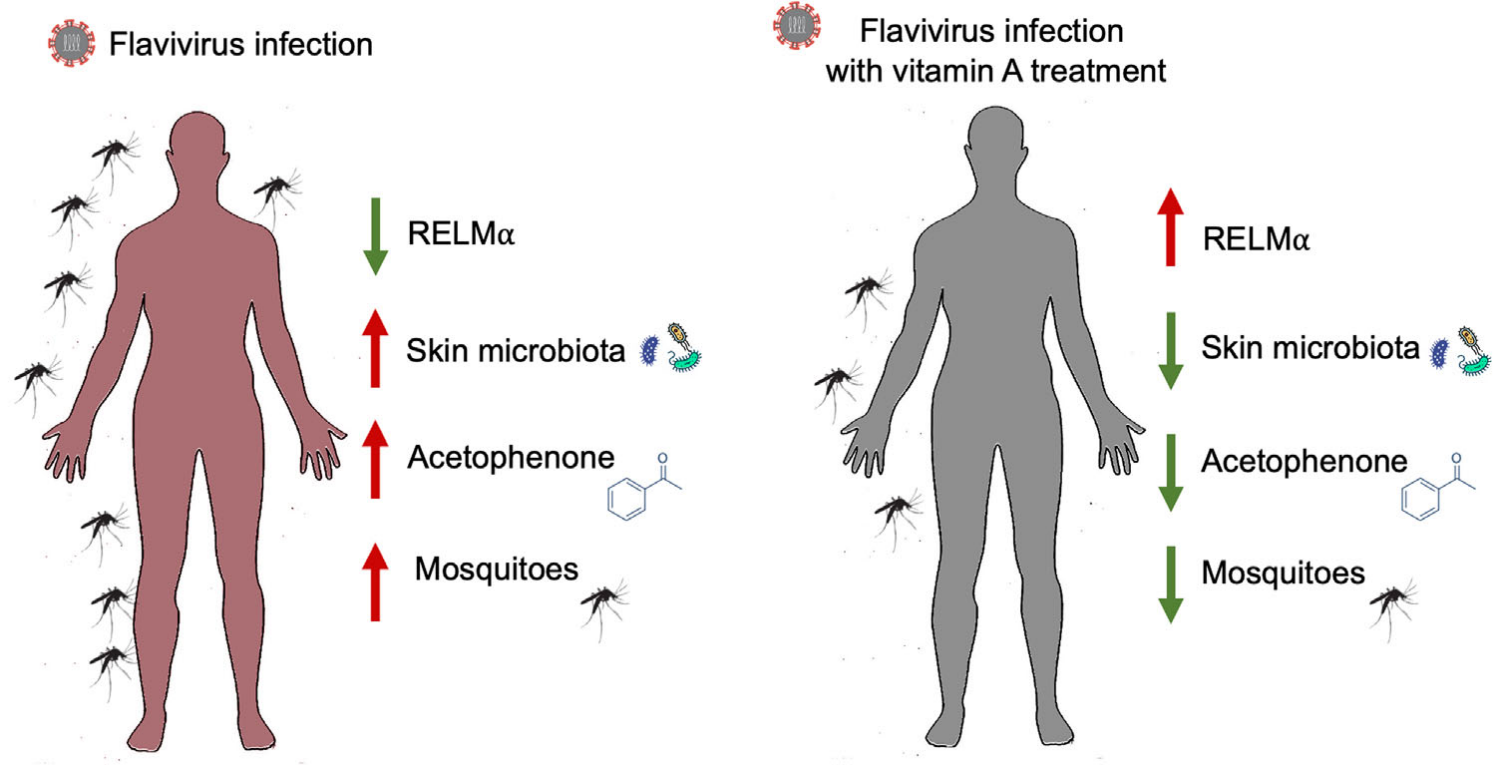
Flaviviruses affect virus transmission via the skin microbiota. Flaviviruses are able to suppress the expression of RELMα, an antimicrobial protein that enhances the relative abundance of acetophenone‐producing bacteria, attracts more mosquitoes and facilitates viral transmission. However, oral administration of vitamin A derivatives to the host induces the expression of RELMα, regulates the skin microbiome homeostasis, decreases mosquito attraction and blocks viral transmission.

Acetophenone released from humans or animals mainly originates from skin commensal microorganisms on the body surface and is a typical bacterial metabolite.^5^ After the commensal microorganisms on the skin were removed, attraction to mosquitoes decreased for virus‐infected mice. Further studies showed that dengue and Zika virus infections led to a significant increase in the abundance of *Bacillus* spp. on the host skin, which are capable of metabolizing and producing large amounts of acetophenone. Thus, the authors revealed that hosts infected by mosquito‐borne viruses attract more mosquitoes. Viral infection increases the proportion of specific bacteria on human skin, which considerably increases the ability of infected individuals to emit acetophenone, thereby significantly enhancing the behavioural propensity of mosquitoes towards infected hosts^4^ (Figure 1).

Subsequently, the authors performed RNA‐Seq analysis of the skin of virus‐infected and noninfected mice. The resistin‐like molecule‐alpha (RELM‐alpha) gene was significantly downregulated in the skin of infected hosts. RELM‐alpha is a major immune determinant factor of host skin microbial homeostasis and is specifically expressed in mammalian keratinocytes and sebaceous gland cells.^6^ Further results by the authors showed that RELM‐alpha and RETN (RELM‐alpha homologue in humans) could efficiently inhibit the proliferation of several skin microorganisms. Previous studies have shown that RELM‐alpha/RETN expression is regulated by the retinoic acid receptor signalling pathway (RAR signalling pathway).^6^ Oral administration of vitamin A derivatives to mice induces the expression of RELM‐alpha and RETN by activating the RAR pathway. The results reported by the authors showed that oral administration of a vitamin A derivative, isotretinoin (a clinically used drug for dermatological treatment), to mice infected with dengue and Zika virus effectively restored RELM‐alpha expression in the skin of infected mice and inhibited the release of acetophenone from infected hosts by suppressing the proliferation of *Bacillus* spp. Thus, after oral administration of isotretinoin to the infected host, the mosquitoes are unable to locate and detect the infected host through the host's acetophenone, thus interrupting virus transmission^4^ (Figure 1). However, the signalling pathways through which flaviviruses cause host transcriptomic changes remain unclear. Further research will enable us to comprehend how viruses interact with host skin microorganisms.

Collectively, these findings reveal the molecular basis of mosquito‐borne virus transmission and offer novel insights for mosquito‐borne virus control. Such foundational research has the possibility of being transformed into disease prevention and life‐saving measures (Figure 1). From a clinical point of view, the modification of host odour as a result of viral infection is a clinically interesting observation. Rapid diagnostic techniques for infectious diseases based on volatilomics deserve further in‐depth study. Compared with traditional clinical diagnostic techniques for infectious diseases, volatilomics‐based diagnostic techniques have the advantages of high resolution, specificity, simplicity and non‐invasiveness.^7^

## CONFLICT OF INTEREST

The authors declare no conflict of interest.
